# The potential of small molecules to modulate glycosylation by media design

**DOI:** 10.1186/1753-6561-9-S9-P38

**Published:** 2015-12-14

**Authors:** David Brühlmann, Anaïs Muhr, Jürgen Hemberger, Markus Sauer, Henri Kornmann, Martin Jordan, Hervé Broly

**Affiliations:** 1 Merck Serono SA, BiotechProcess Sciences, Corsier-sur-Vevey, Switzerland; 2 Department of Biotechnology and Biophysics, Julius-Maximilians-Universität Würzburg, Germany; 3 Institute for Biochemical Engineering and Analytics, University of Applied Sciences Giessen, Giessen, Germany

## Background

A large number of recent publications demonstrate the effect of cell culture media on post-translational modifications of recombinant proteins [1]. In this study, aiming to extend the toolbox of media design beyond the commonly known media components, a large variety of cell culture compatible chemical components such as sugars in industrial relevant Chinese hamster ovary cell lines (CHO) expressing recombinant antibodies were identified and tested. One promising novel supplement is presented hereafter.

## Materials and methods

Fed-batch experiments in duplicates were conducted in 96-deepwell plates at a working volume of 450 µL in each well. Before seeding the culture with either a CHO-S or CHO-K1 cell line at 0.3·106 viable cells/mL and 0.2·106 viable cells/mL, respectively, the cell culture media was enriched with 0-50 mM of oligosaccharide. Bolus feeds were added every two or three days. Viable cell density was measured by Vi-CELLTM(Beckmann Coulter) throughout the culture and at the end of the culture at working day 14,product titer was determined by Octet® (fortéBIO).The supernatants of each culture were purified and N-glycan analysis was performed by CGE-LIF technology (Life Technologies).

## Results

Low and intermediate concentrations of the oligosaccharide (0.1-10 mM) tended to increase the viable cell density of the CHO-S cell cultures. At 30 mM and 50 mM of oligosaccharide cell growth was strongly reduced. The peak cell density amounted to 4·106 viable cells/mL while the control cultures reached 10·106 viable cells/mL. The CHO-K1 cell cultures exhibited comparable cell growth with respect to the control up to 30 mM of supplement. At 50 mM a slight decrease of the viable cell density was observed. Both cell lines yielded comparable amounts of recombinant antibody at harvest on working day 14 up to 10 mM of supplement. Beyond this concentration product titer decreased. At a concentration of 50 mM, harvest product titer of CHO-S cell cultures decreased to 61 % of the control culture titer. CHO-K1 cultures showed a greater tolerance to the oligosaccharide presence. Hence, the titer at 50 mM still amounted to 76 % of the control.

**Figure 1:**
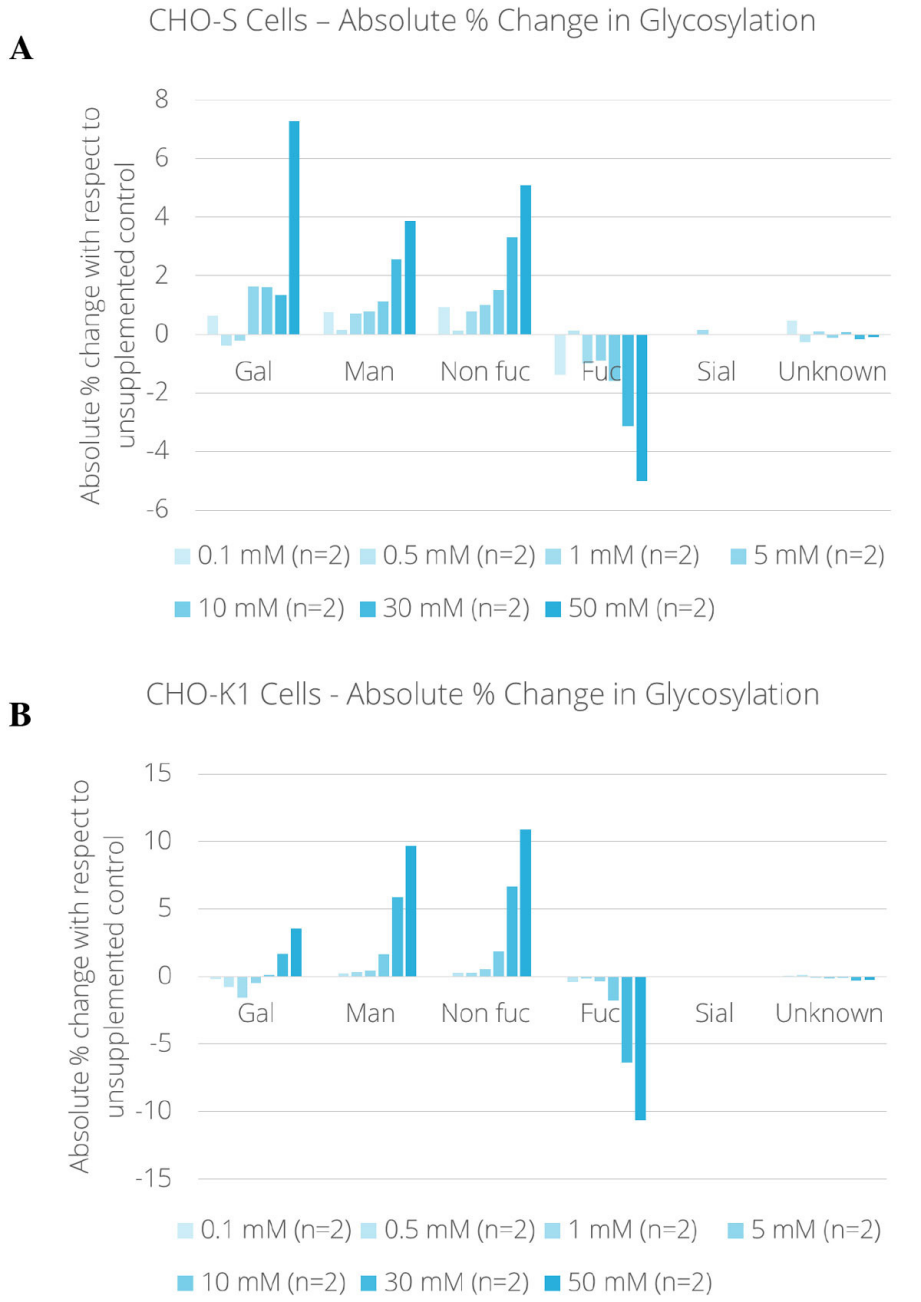
**Absolute % change in glycosylation in function of carbon-source supplement concentration (0**.1-50 mM) with respect to non-supplemented control. **(a) **CHO-S cell culture. (b) CHO-K1 cell culture.

Figure 1 presents the absolute percentage change in glycosylation in function of the concentration of the added concentration oligosaccharide in the culture medium prior to inoculation. The glycosylation changes are expressed in absolute percentages: % glycan(x mM supplement) - % glycan(control) = absolute % change.

The abundance of galactosylated species slightly decreased at supplement concentrations from 0.1 to 1 mM in both CHO-S and CHO-K1 cell cultures. At higher concentrations, galactosylation was favored and thus increased by 7.3 % (CHO-S), and respectively, 3.6 % (CHO-K1) at 50 mM. The increase of high mannose species (Man5, Man6 and Man7) correlated with the supplement level in the medium of each culture. As shown in Figure 1A, at low supplement concentrations in CHO-S cell cultures, high mannose species increased by 0.7 %,at 30 mM by 2.6 %, and at 50 mM by 3.9 %. Figure 1B displays that with this approach a stronger increase of high mannose species was obtained in CHO-K1 cell cultures while limiting the impact on cell growth even at the maximum tested supplement concentration. Up to 5 mM, no substantial increase of high mannose species was obtained. However, at 10 mM, with comparable viable cell density and titer with respect to the control, the high mannose level was 1.7 % higher than the control. By further raising the supplement concentration to 30 mM and 50 mM, the generation of high mannose species became more abundant. At 30 mM the increase amounted to 5.9 % and at maximum concentration to 9.7 % with limited impact on cell growth and as described above a titer reduction of about 25 %.

As a consequence of the increase of high mannose species, a fucosylated species increased and fucosylated species decreased. No important impact on sialylated and unknown glycan species was observed in our experiments.

## Conclusions

Our experimental data demonstrate the feasibility and the great potential of protein quality engineering by cell culture media design. Rather than modifying the gene expression of the cell line, including knockout techniques for changing the host cell type, media design is an attractive alternative to modify the glycosylation profile as it allows to rapidly tune the quality profile within the cell's potential. Media optimization is hence expected to become a commonly used strategy to enhance the pharmacological properties of tomorrows' therapeutic molecules.
